# Policy, law and post-abortion care services in Kenya

**DOI:** 10.1371/journal.pone.0204240

**Published:** 2018-09-21

**Authors:** Michael Mbithi Mutua, Lenore Manderson, Eustasius Musenge, Thomas Noel Ochieng Achia

**Affiliations:** 1 African Population and Health Research Center (APHRC), Nairobi, Kenya; 2 School of Public Health, University of the Witwatersrand, Johannesburg, South Africa; 3 Institute at Brown for Environment & Society (IBES), Brown University, Providence, Rhode Island, United States of America; 4 Centers for Disease Control and Prevention, Nairobi, Kenya; Indian Institute of Public Health, INDIA

## Abstract

**Background:**

Unsafe abortion is still a leading cause of maternal death in most Sub-Saharan African countries. Post-abortion care (PAC) aims to minimize morbidity and mortality following unsafe abortion, addressing incomplete abortion by treating complications, and reducing possible future unwanted pregnancies by providing contraceptive advice. In this article, we draw on data from PAC service providers and patients in Kenya to illustrate how the quality of PAC in healthcare facilities is impacted by law and government policy.

**Methods:**

A cross-sectional design was used for this study, with in-depth interviews conducted to collect qualitative data from PAC service providers and seekers in healthcare facilities. Data were analyzed both deductively and inductively, with diverse sub-themes related to specific components of PAC quality.

**Results:**

The provision of quality PAC in healthcare facilities in Kenya is still low, with access hindered by restrictions on abortion. Negative attitudes towards abortion result in the continued undirected self-administration of abortifacients. Intermittent service interruptions through industrial strikes and inequitable access to care also drive unsafe terminations. Poor PAC service availability and lack of capacity to manage complications in primary care facilities result in multiple referrals and delays in care following abortion, leading to further complications. Inefficient infection control exposes patients and caregivers to unrelated infections within facilities, and the adequate provision of contraception is a continued challenge.

**Discussion:**

Legal, policy and cultural restrictions to access PAC increase the level of complications. In Kenya, there is limited policy focus on PAC, especially at primary care level, and no guidelines for health providers to provide legal, safe abortion. Discrimination at the point of care discourages women from presenting for care, and discourages providers from freely offering post-abortion contraceptive guidance and services. Poor communication between facilities and communities continues to result in delayed care and access-related discrimination.

**Conclusion:**

Greater emphasis should be placed on the prevention of unsafe abortion and improved access to post-abortion care services in healthcare facilities. There is a definite need for service guidelines for this to occur.

## Background

Across Africa, one in every ten maternal deaths is from complications related to unsafe abortion [1]. Post-abortion care (PAC) is designed to minimize morbidity and mortality associated with unsafe abortion by addressing incomplete abortion and treating complications, and to prevent recurrence, linking women to other reproductive health services, and providing them with post-abortion contraception [2][3,4].

Kenya’s maternal mortality ratio (362 per 100,000) is highest among women of peak reproductive age (25–39) [5], and in this group, up to 17 percent of deaths may be associated with induced abortion [6]. The incidence of unsafe abortion in Kenya is among the highest in Africa [7], with the majority of women presenting at healthcare facilities with between moderate and severe complications as a result. Such cases often require specialized treatment and exert pressure on already stretched human and material resources for health.

Policy and legal contexts shape the quality of services provided; these are international, regional and country-specific in content or implementation [8,9]. Abortion remains an illegal or highly restricted and emotive issue in most countries globally [9,10]. Limited access to safe abortion, both formally and informally, increases women’s recourse to unsafe termination procedures, and negative community and facility-based attitudes limit the effectiveness of interventions to provide quality PAC services. Although legalizing abortion alone may not reduce the incidence of abortion, it reduces women’s resort to unsafe terminations and delays in seeking care, and increases service provider willingness to provide care [11]. These factors, achieved through improved cultural acceptability of abortion [12], combine to reduce complications associated with unsafe abortion, while also increasing equity in access [13]. Such policy initiatives must be accompanied by proper community involvement to demystify abortion and raise the awareness of services, including post-abortion care. Women are often profoundly reluctant to seek such care, even where abortion is legal, due to cost [14] and lack of information on the law [15]. The legalization of abortion in the recent past, such as in Ghana, South Africa, and Ethiopia, has been pivotal in reducing morbidity and mortality, but the need for complementarity of approach has been underscored [13–15]. Whether or not abortion is legal, women will use available means to terminate unwanted pregnancies and therefore, the delivery of quality PAC services at all levels of care prevents severe morbidity and associated mortality [16].

In Kenya, where this study was conducted, constitutionally, abortion is illegal unless, in the opinion of a qualified medical practitioner, the health or the life of the mother is at risk or unless permitted by any other law [17]. Furthermore, there are conflicts between the constitution, which was passed in 2010 and theoretically allows abortion, and the penal code, which criminalizes abortion [18]. These contradictions discourage women from seeking care, shape service providers’ perceptions of abortion, and influence facility capacity to deliver PAC. Restrictive access to safe abortion care, because of legal prohibitions and cultural barriers, is known to increase the severity of complications from abortion. Under such restrictive policy and legal environments, women resort to abortions performed outside of clinical services in unsafe conditions and by unqualified providers [19].

To ensure quality care, PAC needs to be patient-centered, and the service provider needs to feel protected [20]. In addition, designing healthcare policies that aim at improving the quality of provider-community interactions encourages the early detection of pregnancy complications, regardless of cause, and enables an efficient community referral system to reduce the risks of delayed care seeking [21,22].

The quality of PAC framework was introduced globally over 25 years ago, and focuses on five distinct areas of intervention to ensure quality: equipment, supplies, and medication; use of appropriate technologies in the management of complications; technical performance; interaction between women and service providers or staff; and information and counseling [23]. This framework recognizes the need for correct equipment and medications to ensure safety and effectiveness of recommended PAC procedures [2]. Functional equipment for abortion care, sterilization, maintenance, and resupply are essential to enable facilities to deliver PAC at all times [24,25]. The use of appropriate technology rather than outdated techniques such as sharp curettage (Dilation and Curettage) improves quality PAC [26,27] and patient satisfaction with these services [28,29]. Adopting appropriate technologies can also reduce hospital stay, the cost of treatment and strain on resources, and increase the safety of PAC services [30]. This must be complemented by provider willingness to adopt these technologies [31]. Essential supplies, including pain medication and antibiotics, improve patient experience with care [32], leading to positive perceptions on quality of care, so increasing community trust in services offered and prospects of future utilization [11,33–35]. Contraceptive commodities and their continued availability reduce risks of further unintended pregnancy. Ideally, these interventions should be complemented by infrastructural improvements, such as dedicated abortion care clinics to address abortion patient needs without discrimination [15,36,37]. Such interventions appear to reduce the demand for clandestine abortion terminations, which occur even when abortion is legal [38].

Strong service provider skills are essential in delivering quality PAC at all available levels of care. This includes expanding the roles of different service providers and building the capacity of lower cadres of care, including nurses and midwives [39,40][25][41,42].

To guarantee quality PAC, interactions between service providers and patients must ensure confidentiality by establishing an atmosphere of trust and empathy and respecting the patient’s rights [3,43]. Patient-centered care dictates the need to understand the specific needs of individual women, including future fertility intentions, contraceptive preferences and past experiences with care. Information must be provided in a manner that enables the patient to make informed choices, which includes a treatment plan and social support to patients [44,45].

In this article, we consider the contribution of the legal and policy context on quality of PAC in healthcare facilities in Kenya, as described by service providers and patients. We begin by exploring some of the policy-level factors that affect the access to and delivery of quality PAC services, focusing on dimensions of quality care as described in the PAC quality framework [3,23,46–48].

## Methods

### Study design and setting

The study on which this article is based was cross-sectional, using in-depth interviews to collect qualitative data. Public and private “high volume” healthcare facilities, including general and referral hospitals at Levels 4, 5 and 6, were selected, based on the number of PAC cases reported in the facilities in the most recent data [7,49,50]. Public facilities are owned and run by the government, and private facilities are managed by individual proprietors, non-governmental organizations or faith-based organizations. In Kenya, the Ministry of Health, under the Kenya Essential Package for Health (KEPH), defines six levels of preventive and curative health services, both for public and private facilities, ranging from level 1–6. Level 1 is the lowest level, and forms the foundations of service delivery at the community level; it includes village health committees and community health units. Levels 2 and 3 (dispensaries, health centres, and maternity/nursing homes) offer promotive, preventive, and curative services. Levels 4 and 5 (primary, secondary and tertiary hospitals) offer curative and rehabilitation services, with a limited number of preventive/promotive care programs, while level 6 is the highest level of care, comprising the national referral hospitals. Sampling for this study was purposive to include only facilities that managed a high number of PAC cases, so allowing us to recruit sufficient cases over a short period (one week for data collection) when trained qualitative interviewers were able to work at each facility.

### Sampling

Sixteen hospitals in three regions − Nairobi (5), Central (5) and Eastern (6) − were sampled purposively by regional area, level, and reported quality of care. These facilities were classified into “high,” “medium” or “low” quality [37] to ensure representation of facilities at different levels of care and to gain a wide view of provider and patient experiences according to the facility level. This categorization was based on data from an earlier study from which these facilities were sampled. The study used the essential elements of PAC framework to categorize all participating facilities into three categories above (for details, see Mutua et al., 2017)). To achieve greater diversity and in-depth opinions on care from patients we purposively selected patients from a sub-sample of six of the 16 facilities, two from each of the three levels of quality stated above. This selection was mainly based on facility distance from Nairobi and size, with the intention to focus this selection on high-volume facilities able to provide sufficient numbers of interviews within a short period. Five patients in each of the six facilities were recruited and interviewed, together with one service provider at each of the 16 facilities. This yielded a sample of 30 patients and 16 provider interviews. However, due to a low number of patients in some facilities, only 21 patients were interviewed of the targeted 30 patients. All service providers were interviewed: of the 16 interviewed, eight were nurses and eight clinical officers.

### Data collection

Two interview guides were developed and reviewed by the lead author and all co-authors. The guides were structured specifically to encourage discussions with service providers and patients on their understanding of the different components of the quality of PAC framework. The service provider interviews lasted on average 34 minutes while patient interviews lasted 21 minutes. Both provider and patient interviews were conducted in a private room in the health facility to ensure maximum privacy of information shared. All interviews were conducted either in English or Kiswahili, and transcribed and translated into English if the interview was conducted in Kiswahili. Field data collection was conducted between May and July, 2017.

### Data

The main PAC service providers in selected facilities were recruited and interviewed by one of a team of three experienced and well-trained qualitative field researchers (all female). Women who were treated for PAC in selected healthcare facilities were interviewed after discharge, prior to their return home. The principal investigator (first author, PI, male) trained and worked with the field researchers to ensure that they had a good understanding of the study objectives and were mindful of its ethics. Interviews were adapted throughout the process through a constant review of data collected. All interviews were audio-recorded, and quality control and improvement were ensured through listening to recordings and discussions for providing feedback to interviewers. Once each interview was completed, it was forwarded electronically to a central office and archived on a password-protected computer, accessible only to the PI. Once all data were received, a transcriber worked with the PI; she was well trained to understand the survey objectives and the ethics of handling sensitive data. All data were transcribed into Microsoft word and as required, translated from Kiswahili into English. All patient and provider identifiers were replaced with codes to minimize the risk of identification of patients during data coding and analysis.

### Survey ethical consideration

Ethics approval was obtained from the Ethical and Scientific Review Council of African Medical Research Foundation (AMREF), the Kenyatta National Hospital (KNH) and University of Nairobi (UoN) Ethical Review Committee (ERC), and the Human Research Ethics Committee (Medical) at University of the Witwatersrand. Survey approvals were also obtained from the Kenya National Commission for Science, Technology, and Innovation (NACOSTI) and the Ministry of Health. Written informed consent as approved by AMREF were obtained from each participant before any interview was conducted. All minors who were already securing PAC services were deemed to be independent and able to provide their own informed consent, without parental consent.

### Analysis

Data were analyzed deductively, using a predefined set of themes from the essential elements of PAC framework. Additionally, inductive analysis was used by identifying any additional themes on quality of care at the facility as described by the providers and patients. A double coding approach was used to compare independent codes between a trained and experienced coder and the PI, and an agreement was built on the codes before final coding in NVIVO. Final analysis involved in-depth interrogation of the data for both visible and underlying meanings assigned to quality of care from the perspectives of the service providers and patients. Specific verbatim quotes were extracted from the data, which express respondent views concerning certain propositions of quality care. Below, we identify people only by broad class (provider, patient) when we quote them directly.

## Results

In this section, we first discuss the legal and policy issues that frame the provision of quality PAC services in healthcare facilities in Kenya, by exploring all issues related to the abortion law in Kenya and the policy environment within which PAC services are sought and offered within healthcare facilities. We then discuss the different components of the essential elements of PAC framework, and how PAC services are sought and delivered in the context of the above legal and policy environment.

### The constitution and the law

For fear of legal repercussions, patients often fail to disclose their abortion history, withholding information from providers on the method used to induce an abortion, for instance, and estimated gestational age. Under such circumstances, especially when physical examination is also misleading, inadequate disclosure can result in inaccurate management and impedes the use of appropriate technologies, with potential risks to the woman:

In the process of taking the history, there is another challenge because a lady can decide not to tell at which gestation stage the child was. You can say my last menstrual period (LMP) was the previous week yet you find at the expulsion of the fetus you see the fetus was already developed. (Provider, level 4, private, low quality facility)

### How policy is derived from law, and how it is translated

In 2016 changes in law led to the registration and inclusion of Misoprostol and Mifepristone as essential drugs for the management of obstetric and gynecological indications, resulting in increased access of these as abortifacients. This significantly reduced the use of crude means of abortion termination (such as the insertion of a sharp object, ingesting concoctions or heavy massage). However, some service providers found challenges with the unregulated access of these drugs, often without proper controls on drug prescription and usage:

You find that abortifacients could not be sold in chemists without a doctor's prescription. Nowadays they are very available, even a standard 8 girl can go to town, purchase the abortifacients, come here bleeding in shock … the drug is now an over-the-counter drug. (Provider, level 5, public, high quality facility)

The organization of healthcare services from national to county to facility level poses additional restrictions to quality PAC access. Processes such as the acquisition of appropriate equipment and medication, integral to quality PAC, are managed by healthcare administrators who often lack requisite medical skills:

You are dealing with someone who is not a medical person in the procurement department. You are dealing with a matron that is not interested maybe” (Provider, level 4, public, low quality facility).

There was a lack of guidelines on the provision of PAC and legal abortion care in all facilities and at all levels. In the absence of such clarity, some service providers opted to provide safe abortion services at night, but during the day, these cases would be recorded and managed as PAC cases. Service providers emphasized the need for interventions by county and national health management officials to provide effective guidelines for abortion care and PAC; guidelines, they felt, would improve their confidence to provide PAC, because they offer clarity in relation to the law:

I think in the county we have the RH (Reproductive Health) department. They are doing a good job but mostly they are concentrating on the family planning. In the county government, the RH department should really concentrate on how to reduce cases of aborting and on how to increase awareness on PAC. (Provider, level 4, private, medium quality facility)

Service providers acknowledge the importance of collaboration between different RH actors. Despite the cost implications of such ventures, service providers identified the need for service collaboration between county governments and other RH stakeholders to support identifying and addressing existing quality gaps in PAC services.

### Delayed care due to limited facility capacity

Lack of requisite capacity to offer quality PAC in facilities resulted in women receiving “partial referrals” for certain services such as radiological examinations and surgical obstetric or gynecological care. These referrals extended the duration of treatment and increased the risks of excessive bleeding and untreated infections, causing concern among patients and service providers alike. Lack of ultrasound equipment in most level 4 facilities, for example, meant that patients were regularly referred to other facilities for obstetric or abdominal scans, after which they were then to present back to the referring facility for further management. The need to go to a different facility for a scan led to delayed intervention and increased the risk of severe complications. As one service provider in Central Kenya emphasized, less time was lost when patients were referred to a single facility for both the scan and completion of treatment. However, even when this was possible, in some cases patients were asked to return to the referring facility because of heavy workloads and lack of space in the larger facilities:

Our nearest referral hospital is [Facility A] level 5, where they usually can go and first do the scanning, if they wish to complete their procedures there they can but some of them due to the workload in [Facility A], after scanning they usually come back here, so we do still the MVAs here. If it is septic we have the wards and thus we admit them. (Provider, level 4, public, high quality facility)

Patient referral from smaller facilities was common. In the larger facilities, patients could wait overnight to be seen by a doctor. For example, one patient was referred to a facility, having already expelled one of a twin fetus; the second fetus was only partly expelled. Overnight, this patient remained under ineffective pain killers, awaiting a doctor to perform an evacuation.

In smaller level 4 facilities, patients often needed specialized surgical interventions, which only a specialist gynecologist could perform. Yet a referral was often not feasible, due to the long distance between facilities and transfer costs. Some of these facilities lacked ambulances with advanced life support (ALS) to ensure patients of a seamless transfer to a level 5 hospital. Even for facilities with ALS ambulances, certain complications such as postpartum hemorrhage and ruptured uterus could still result in death of the woman while in transit.

Patient referral and transfer gaps were evident, with providers experiencing difficulties to identify the right facility to which they could refer patients. This caused delays, leading to severe complications and occasionally the death of a patient:

You might find that when you are referring a patient there could be some difficulties you encounter before the patient can be accepted in the facility where you are referring her. So they should try and harmonize in terms of linking to the system. ~ (Provider, level 4, public, medium quality facility)

Infection control prevents sequelae from unsafe abortions and must be controlled to prevent future pregnancy loss, and pain medication is important to ensure patient-centered care. However, lack of infection-control medications including antibiotics was common, as was the lack of analgesics. Patients were often referred to facilities such as local chemists to purchase these medications; others endured painful procedures without effective analgesics. Other health facilities referred patients because of the lack of blood or blood products, or lack of capacity or equipment to cross-match blood or transfuse. Commonly, there were insufficient instruments and lengthy instrument sterilization procedures, which with high numbers of patients hindered service provision and increased the number of referrals.

Privacy is essential for a conducive environment for patients to openly discuss their condition, for informed management of their complications, and to discuss their reproductive history and aims. However, patient admission facilities were often insufficient, resulting in patients having to share beds, premature patient discharge, and referrals to other facilities. In addition to infringements of patient privacy and right to confidential treatment, there was evidence of lack of capacity by lower level facilities to manage the number of cases that presented for PAC at these facilities. One patient had been admitted to three other facilities before a fourth admission at a level 5 hospital, and by the time she was admitted, she was unconscious. Many facilities also lacked sufficient evacuation procedure rooms, especially when a service provider was required to manage more than one patient at a time. As a result, PAC procedures were often conducted in maternity wards, despite concerns from providers of the potential psychological distress to patients by combining patients who had lost pregnancies with those who had successful births.

### Technical performance

Lack of clarity on abortion law, and lack of care guidelines, impacted negatively on the capacity of service providers to provide quality PAC. Some patients were critical of the quality of support they received from staff at health facilities in relation to surgical and medical care, counseling and family planning advice. Repeat procedures, often the result of incomplete evacuation due to inadequate provider skills, were common. According to patients, these “revacuations” were mainly due to provider’s inability to make a specific diagnosis, and this increased the costs of care to both the patient and the health system.

Staff turnover, due to transfer of trained personnel between facilities, also impacted on quality PAC. While transfers can improve the capacity of other facilities to offer PAC, within facilities, necessary transfers demotivated other providers and caused confusion among patients. According to service providers, skill improvement lacked a focus on the capacity to prevent patients’ risk of repeat abortion:

To many people, the knowledge is limited to just provide what it takes to take this fetus out and then the other things is not important. The only important thing is that the pregnancy is out. For me, there is that gap. I think it is a real discrepancy because tomorrow they will go get pregnant again and like that. (Provider, level 4, public, low quality facility)

### Interactions between women and service providers or staff

Uncertainty around the legality of abortion often led to patient discrimination at healthcare facilities. Service providers often condemned abortion and discriminated against women who secured abortion, and did not always support patients’ healthcare needs. In some cases, they designated duties such as within hospital transfers to patients or their caregivers, causing delays in care provision. Providers were often unsupportive and unresponsive to the patients’ needs, and failed to treat them with courtesy. According to patients, providers unreasonably withheld guiding them through the facilities. One patient described how two or more patients used one stretcher, without measures to avoid infection, equally exposing patients and caregivers to infections:

Yes. Then we came with the stretcher, they returned us and told us that it wasn’t the stretcher for casualty, but by good luck there was a patient that was being discharged and that is when we took the stretcher; it was just by chance. (Patient, level 6, public, high quality facility)

Some providers also spoke of disrespect to patients (from providers other than themselves) and argued the need for positive attitudes by health providers to encourage timely seeking of PAC services. Young women were particularly affected by negative attitudes, from providers who believed that they should not have been pregnant in the first place. These attitudes have also been associated with the high incidence of unsafe abortion in the country, and severe complications when women opt to self-medicate complications from unsafe terminations. In addition to age, discrimination occurred on the basis of marital status, with service providers likely to conclude that an abortion was induced if a patient was unmarried. Unmarried patients were therefore more likely to lie about their marital status, in order to gain access to the same level of care as provided to married patients.

### Women are discouraged from accessing care and delay doing so

Quality care requires the continued availability of PAC services, and any service disruption can lead to life-threatening complications or result in lifetime consequences on patients’ reproductive and psychological health. During the study period, however, there were recurrent service disruptions due to industrial disputes: a national doctors’ strike lasted over three months; a national nurses’ strike for six months; and a clinicians’ strike for one month. Holidays and weekends, when the few available providers were off-duty, further increased the number of days when services were unavailable. In addition to delays in seeking care occasioned by legal challenges, as discussed earlier, other forms of service unavailability and stigmatization influenced general patterns of healthcare seeking. Women were aware of service disruptions, and they often presented to public facilities only when their conditions were too grave for further self-management and when they could not afford private facilities. Furthermore, most PAC cases in Kenya are treated in public facilities [7]. Even during the strike period, most public facilities always had nurses on duty, but care was delayed when the nurses were unable to perform a particular procedure or the procedure was not the nurse’s responsibility as set out in the service guidelines and Ministry of Health regulations:

Because if you come here at night, there is no doctor, there were just nurses, there are some things that the nurses can’t do like that one of mine they were telling me that I had to wait for the doctor, what if it was something serious that could kill me? (Patient, level 6, public, high quality facility)

### Can women afford to access PAC?

The essential elements of the PAC framework do not explicitly clarify cost of care, whether direct or indirect. In Kenya, healthcare services are not free; they are subsidized in public facilities but not in private facilities. In cases of emergencies at night, or during weekends and holidays, most primary care public facilities are closed, and patients travel for long distances to reach an available secondary and tertiary facility. In such cases, these patients would seek care in private facilities, but these services are not generally affordable. Patients who could not pay for the services in private facilities would be referred to public facilities, while those in public facilities would miss certain services or procedures until they can raise funds to pay for these services. Others were discharged without receiving critical treatment procedures, owing to non-payment. This exposed patients to further complications, such as septic abortion when a patient could not afford to pay for antibiotics, or a perforated uterus for a patient who was delayed before and evacuation procedure.

Regardless of ability to pay, the Kenyan constitution guarantees every Kenyan of the right to emergency care. However, patient referrals increase the out-of-pocket cost of treatment, particularly when referred from public to private facilities. For other patients, the requisite mode of payment for services posed additional challenges. Lack of payment options for patients necessitated the emergence of brokers, who would assist patients to make payments at a fee. Patients were often required to make cash payments for procedures, including large sums of money for surgical procedures. This requirement would restrict services even to patients who could make these payments using an alternative payment mode such as a credit or debit card. In large government hospitals, use of cash was restricted, as facilities favored mobile money transfers (M-Pesa), yet patients were not always able to make such transactions. Consequently, PAC patients were at times required to walk for long distances in search of mobile money services:

Maybe you have cash and not in M-Pesa [mobile money], when you are sent to buy drugs or pay for something, you are told that you should pay through M-Pesa, now with that it delays because you have to move from there and go to M-Pesa, deposit the money so that you can then come back, and pay. If it was someone in a critical condition, he/she can just die.”~ (Patient, level 6, public, high quality facility)

The requirement that patients paid prior to each procedure restricted access to timely care within facilities. Similarly, inability to pay for services led to multiple referrals, with patients typically moving from a private facility near their residence to a public facility, far from their residence. In private facilities, providers reported that patients often could not afford services at all, even after the services had been rendered. In addition to direct treatment costs, even in public facilities where all services are supposedly free, other indirect costs such as toiletries and bathing accessories such as bath basins further increased their out-of-pocket expenses.

## Discussion

PAC services in Kenya continue to suffer significant quality drawbacks owing to the legal and policy environment under which both service providers and patients operate, so impacting on access and provision [51]. There is no clarity of law for either the patient or service provider, and there are currently no guidelines to provide legal safe abortion in Kenya. This in effect means that patients and service provider communication is shaped by fear, with each party worried about legal repercussions, as established in other countries with similar restrictive abortion laws [52]. Fear further fuels discrimination against patients by service providers, and community discrimination against service providers. This leads to delays in seeking care on one hand, and provision of care on the other, which ultimately increases the risks of severe complications among PAC patients.

Abortion in Kenya remains restricted, and there is evidence of high incidence of unsafe abortion [19,50]. Lack of clear guidelines on the provision of safe legal abortion and post-abortion care complicates access to quality PAC in healthcare facilities. Where such guidelines are either unavailable or unclear, career security and personal abstraction of social norms, rather than the need for patient-centered care, formed the basis of the decision-making by most service providers we interviewed [53–55]. However, when providers have full legal backing, quality care can be assured across facilities and its implementation can also be assessed for continued quality improvement. Despite a constitution that guarantees every patient of universal access to emergency care, the ambiguity of abortion law in Kenya contributes to the risks of unsafe abortion, through delayed care-seeking and emergency service delivery [52].

Service providers require full disclosure, including pregnancy gestational age, for a proper choice of treatment option. However, the illegality of abortion creates fear among patients, leading to incomplete disclosures of their history, leading to misdiagnosis and use of inappropriate technology in patient management [24], as has been previously demonstrated [37]. The entry of abortifacients in Kenya has significantly reduced the use of crude means of pregnancy termination, yet this alone cannot solve the challenges of unsafe abortion [51]. These abortifacients have other functions in care, especially in treating post-partum hemorrhage, which necessitate their registration in the country as essential medical drugs. However, as service providers pointed out, their over-the-counter access may not guarantee safer abortions unless coupled with better controls for their administration.

There is compelling evidence that effective post-abortion contraception reduces the risk of repeat abortion among an already exposed group [56–58]. However, facilities owned or managed by faith-based organizations restricted access to contraceptive services. Consequently, patients were referred to other facilities for post-abortion contraceptives, although such referrals are known to be highly ineffective due to low, incomplete or inconsistent follow-up.

Availability of essential drugs and other consumables that are critical to care delivery, such as disinfectants and pain medications, is restricted, and service providers have minimal controls over such decisions [35]. Pain medication and infection control were equally deficient and in most cases, patients complained of either lack of these medications in public facilities or their ineffectiveness. Effective pain management is an essential component of care, which assures patient satisfaction with care and future service utilization. However, providers were rarely involved in procurement processes or in discussions on sanitation and sterilization of surgical facilities.

Government or facility policies on the recruitment and exposure of facility workers to critical procedures such as infection control and procurement decisions further deterred access to quality PAC. When healthcare management guidelines charge such non-medical or insufficiently trained staff with these responsibilities, care decisions are based on reasons other than quality care, such as cost-saving and budgetary controls.

There was evidence of deficient interaction between women and service providers as well as negative attitudes towards women. At some facilities patients were denied services to avoid being branded as “abortion clinics” in the communities. Service providers in such facilities often resorted to quick referral for services, even when they understood the need for timely PAC and the consequences of denying or delaying such services. According to service providers, community engagements geared towards changing the perception on abortion would create a favorable environment for PAC delivery. Patient discrimination and fear of facility-based discrimination resulted from negative attitudes towards abortion from both service providers and community members. While some providers withheld services, especially contraceptives to adolescents and young women, others denied women critical nursing care, forcing them or others caring for them to perform roles that would normally be performed by providers. There were also evident provider training gaps which impacted on patient safety and service quality, as highlighted by some of the providers and patients interviewed (see also [59]).

The treatment of incomplete abortions and complications from abortions rested on the level of healthcare facility preparedness and the capacity of service providers to offer quality PAC. Lack of responsiveness to patients and inadequate patient support significantly affected patients’ perception of the quality of PAC services. Emergency care was significantly impeded by the unavailability of the right service providers for certain procedures, especially doctors, and during the night, weekends or public holidays. These capacity gaps were also evident in the number of patients who required multiple evacuations, often leading to longer hospital stays and increased the cost of care to both the patients and the healthcare system. There were also quality gaps in patient counseling services in most facilities, despite evidence of the role of effective counseling on the future contraceptive behavior of PAC patients [60–62]. The need for strengthening the capacity of lower service provision personnel such as nurses, midwives and clinicians is evident in resource strained countries like Kenya where it is not possible to guarantee a medical doctor or a specialized obstetrician/gynecologist at all times [63]. Similarly, frequent transfer of medical personnel often disrupted services in these facilities.

Timely service delivery minimizes the risks of post-abortion complications. Between facilities, as in other recent studies, long and unspecific referrals delay care, with patients often experiencing multiple referrals due to a shortage of bed space, equipment or technical skill by service providers [64], similar earlier findings on delays in making critical decision to transfer patients [65]. Lack of facility capacity, especially unavailability of the requisite equipment and space, was prevalent and resulted in partial or full referrals. Such patients spent more time seeking care, leading to higher treatment cost [66]. Most patients preferred private facilities over public facilities due to the long waiting time at the latter, although they often could not afford such care [19,65,67].

Cost of care was a major barrier to PAC service access [68–70]. Commonly during treatment, patients incurred unanticipated costs, which either delayed the provision of care or, through extended hospital stay, increased costs. In this study, as illustrated above and described in other studies, patients were at times referred to other public or private facilities for specific procedures [19,71]. Even in public facilities where services were deemed free, hidden costs such as the purchase of toiletries often become apparent to patients upon admission, as also described in a Bangladeshi study [72].

As noted, mode of payments available at facilities was problematic, as some facilities restricted cash payments and insisted on mobile money transfers, while others insisted on cash payments even for large payments such as for theatre procedures. These restrictions reduced service access, even when services were available.

PAC services are not addressed by county or national government health agendas. As a result, there is a lack of integration of PAC and family planning services. According to the PAC framework, there is a clear interrelationship between PAC and contraception. Universal access to effective contraception cannot be attained without ensuring effective contraception for all women treated for PAC [73,74].

For continuous and sustained quality PAC, availability and accessibility, service inequity needs to be addressed. In most cases, service disruptions render access impossible for patients and delays care, especially in public facilities (see also Ziraba et al., 2015). These disruptions due to industrial actions, and the unavailability of services over weekends and on public holidays, expose patients to complications and life-long sequelae. Often, PAC cases present as emergencies and the level of facility preparedness to deal with these conditions determines the patients’ post-abortion health outcomes and subsequent quality of life, including their future fertility [47,55].

A number of partnerships are crucial in ensuring quality PAC. Strong public-private partnerships can improve access by creating linkages between public and private facilities, while linkages with other PAC and RH stakeholders can strengthen the quality of service delivery, especially in primary care facilities. Strengthening knowledge sharing between facilities and other non-governmental actors in PAC has been lauded in other settings for the quality of PAC service delivery, and for reducing the risks of unsafe abortions [75–77].

Private facilities, including faith-based facilities, continue to play a complementary role to the public facilities’ reproductive health services. However, this role is not as efficient, where barriers such as distance to services, denial of services and the cost of care exist, and public facilities must be more accommodative to PAC patients and refocus care delivery towards patient centeredness.

## Conclusion

In Kenya, quality post-abortion care is still deficient in healthcare facilities. Continued restrictive abortion laws inhibit quality improvements that might be possible through provider training and community support for women with unintended pregnancies. Lack of policies and guidelines for the provision of safe and legal abortion care continue to impede service delivery. In addition to availability of well-trained service providers, effective care guidelines have been shown to work well in other aspects of care [78].

Legalizing abortion must be preceded or complemented by quality improvement on existing PAC services and awareness of the risks of unsafe abortion, through the promotion of early management of complications and more responsive emergency care. Ensuring proper regulations on the use of medical abortifacients requires policy intervention geared towards improving usage, without reversing the current gains. The need for improved service delivery is critical, guaranteeing the safety and confidentiality of patients and the consequent confidence of service providers when managing PAC patients.

Continued availability of service providers would not only reduce the illness burden to patients but could be life-saving. Training service providers, together with equipping facilities with radiological capacities for the timely detection of incomplete abortions, could improve the quality of care and reduce costs of services and to patients. The greater involvement of service providers in developing the capacity of support staff could ensure an infection-free healthcare environment and continuous skill improvement through knowledge sharing, especially in smaller facilities, with the aim of reducing the need for long-distance travel for care.

There is need to re-organize PAC service provision for informed decision-making on the availability and readiness of equipment and products for quality PAC, including the maintenance, sterilization and availability of the right drugs. Expanded service delivery, with full PAC at lower-level facilities, can ensure continued and uninterrupted availability of services. Facility-based interventions are also needed to improve women’s access to effective contraception and to address religious and cultural concerns about contraceptives as an unacceptable means of managing unintended pregnancies. Better integration of post-abortion care and family planning will increase post-abortion contraception this in turn will improve the disposition of service providers on patient-centered care and counseling for improved contraception, so reducing reliance on unsafe abortion to limit births. While fostering service integration at specific services, combining maternity care and PAC must be done in a manner that minimizes distress for patients who have lost their pregnancies.

A well-defined linkage between facilities will further reduce delays in care delivery and assure patients of favorable treatment outcomes while expanding service delivery to primary care facilities to reduce multiple patient transfers. Further, creating linkages between facilities and communities can help to reduce the strong anti-abortion and anti-contraception views and increase awareness of the risks of unsafe abortion. Stronger partnerships between healthcare providers, public and private actors, and communities to prevent the risks of unsafe abortion may ensure both patients and providers of long-term health improvement and security from legal repercussions.
